# Mechanisms of carbapenem resistance in endemic *Pseudomonas aeruginosa* isolates after an SPM-1 metallo-β-lactamase producing strain subsided in an intensive care unit of a teaching hospital in Brazil

**DOI:** 10.1590/0074-02760160116

**Published:** 2016-09

**Authors:** Luciana Camila Cacci, Stephanie Gomes Chuster, Natacha Martins, Pâmella Rodrigues do Carmo, Valéria Brígido de Carvalho Girão, Simone Aranha Nouér, Wania Vasconcelos de Freitas, Juliana Arruda de Matos, Ana Cristina de Gouveia Magalhães, Adriana Lúcia Pires Ferreira, Renata Cristina Picão, Beatriz Meurer Moreira

**Affiliations:** 1 Universidade Federal do Rio de Janeiro, Centro de Ciências da Saúde, Instituto de Microbiologia, Rio de Janeiro, RJ, Brasil; 2 Universidade Federal do Rio de Janeiro, Faculdade de Medicina, Rio de Janeiro, RJ, Brasil

**Keywords:** P. aeruginosa, SPM-1, intensive care unit, multilocus sequence typing

## Abstract

Carbapenem-resistance mechanisms are a challenge in the treatment of *Pseudomonas aeruginosa* infections. We investigated changes in *P. aeruginosa* carbapenem-resistance determinants over a time period of eight years after the emergence of São Paulo metallo-β-lactamase in a university hospital in Rio de Janeiro, Brazil. Patients admitted to the intensive care unit (ICU) were screened for *P. aeruginosa* colonisation and followed for the occurrence of infections from April 2007 to April 2008. The ICU environment was also sampled. Isolates were typed using random amplified polymorphic DNA, pulsed-field gel electrophoresis and multilocus sequence typing. Antimicrobial susceptibility was determined by disk diffusion and E-test, production of carbapenemases by a modified-CarbaNP test and presence of carbapenemase-encoding genes by polymerase chain reaction. Non-carbapenemase resistance mechanisms studied included efflux and AmpC overexpression by PAβN and cloxacillin susceptibility enhancement, respectively, as well as *oprD* mutations. From 472 *P. aeruginosa* clinical isolates (93 patients) and 17 isolates from the ICU environment, high genotypic diversity and several international clones were observed; one environment isolate belonged to the *bla*SPM-1 *P. aeruginosa* epidemic genotype. Among isolates from infections, 10 (29%) were carbapenem resistant: none produced carbapenemases, three exhibited all non-carbapenemase mechanisms studied, six presented a combination of two mechanisms, and one exclusively displayed *oprD* mutations. Carbapenem-resistant *P. aeruginosa* displayed a polyclonal profile after the SPM-1 epidemic genotype declined. This phenomenon is connected with *bla*SPM-1 *P. aeruginosa* replaced by other carbapenem-resistant pathogens.

The antimicrobial treatment of *Pseudomonas aeruginosa* infections has predominantly involved carbapenems since the 1980s (Papp-Wallace et al. 2011); then, resistance emerged worldwide (Lister et al. 2009). The mechanisms of resistance typically involve OprD porin loss, overexpression of efflux systems, overproduction of AmpC-type β-lactamase and acquisition of carbapenemase-encoding genes (Lister et al. 2009).

Although carbapenem resistance may result from several combinations of these mechanisms (Rodríguez-Martinez et al. 2009b, Castanheira et al. 2014), metallo-β-lactamase (MβL) production is noted in massive outbreaks, including the international dissemination of resistant strains (Maatallah et al. 2011). Intriguingly, SPM-1 MβL, which was discovered in a *P. aeruginosa* isolate in 2002, has been widely spread throughout Brazil (Toleman 2002); it was found elsewhere, but only after acquisition from Brazil (Cornaglia et al. 2011). Some *bla*_SPM-1_-carrying isolates also produce RmtD, which confers aminoglycoside resistance by 16S rRNA methylation (Doi et al. 2007). *P. aeruginosa* carrying *bla*_SPM-1_ and *rmtD1* have potentially become widespread in Brazilian environments, as suggested by a strain isolated from a river crossing in the city of São Paulo (Fontes et al. 2011). The *bla*_SPM-1_ gene is mostly related to a pulsed-field gel electrophoresis (PFGE) type of ST277 (Silva et al. 2011).

SPM-1-producing *P. aeruginosa* clinical isolates that are exclusively susceptible to colistin were identified among patients admitted to a university hospital in Rio de Janeiro, Brazil, from 1999 to 2000 (Pellegrino et al. 2002). In the present study, we aimed to compare the resistance levels of *P. aeruginosa* clinical isolates detected from 2007 to 2008 with those previously described (Pellegrino et al. 2002) and to characterise the mechanisms of carbapenem resistance.

## MATERIALS AND METHODS

*Study design and intensive care unit (ICU) environment screening* - We conducted the present study at a teaching hospital in the city of Rio de Janeiro, Brazil, from April 14, 2007 through April 14, 2008. Patients admitted to the ICU for at least 72 h were included in a prospective cohort study following patients from admission to discharge. Screening for *P. aeruginosa* colonisation in respiratory secretions and rectal swab specimens was performed at admission, on the third day of hospitalisation and then weekly until discharge. The infection control committee defined patients with infections according to established criteria (Horan et al. 2008). The hospital’s Institutional Review Board approved the study (protocol No. 120/06).

Specimens from the hands of the ICU healthcare staff, sinks, faucets, benches, bed rails, mattresses, top of tables and haemodialysis portable equipment (n = 29) were screened for *P. aeruginosa* isolates. These specimens were collected at two time points: April 1 and April 8, 2008. Handprints from health care workers were made on blood agar. Environmental surfaces were screened with moistened swabs, inoculated onto MacConkey broth (Difco Laboratories, Detroit, MI, USA), incubated at 35ºC overnight and identified as described below.

**Fig. 1:**
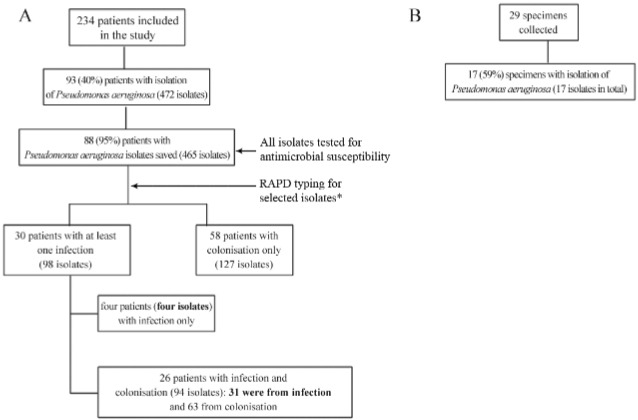
: patient population and bacterial isolates studied. Clinical isolates are shown in A; environmental isolates are shown in B. Isolates marked in bold were selected for analysis of mechanism of carbapenem resistance. *Isolates selected for random amplified polymorphic DNA: all from infection and a sample from colonisation, as described in methods.

*Patient population and P. aeruginosa isolates* - A total of 502 patients were admitted to the ICU; 235 (47%) stayed in hospital for at least 72 h and were eligible for the study. One patient did not agree to participate; therefore, 234 patients were included in the study, as detailed in Fig. 1. *P. aeruginosa* episodes of infections (n = 35) were detected in 30 (13%) of the patients. The most common was bloodstream infection (n = 21), and many of these cases (n = 11) were secondary to ventilator associated pneumonia (VAP, n = 8) and intra-abdominal infection (n = 3). Other primary infections included VAP (n = 8), vasculitis (n = 3), and tracheal, intra-abdominal, and urinary tract infection (one each). A collection of 465 *P. aeruginosa* isolates was recovered from patients and saved, and 17 isolates were obtained from ICU environment and saved.

*Bacterial identification* - Clinical and environmental isolates were grown on MacConkey agar and incubated at 35ºC for 48 h. Blood samples were inoculated into BacT/ALERT (BioMerieux, Askim, Sweden) bottles and subcultured onto blood agar and chocolate agar. Gram-negative bacilli, oxidase-positive, non-fermenters, and motile isolates and isolates capable of arginine decarboxylation were presumptively classified as *P. aeruginosa*. Identification was confirmed by MALDI-TOF as previously described (Bizzini et al. 2010). All isolates exhibited a score value greater than 2.0, which guarantees a reliable species identification. Isolates were stored as suspensions in a 10% (wt/vol) skim milk solution containing 10% (vol/vol) glycerol at -20ºC.

*Antimicrobial susceptibility* - Susceptibility tests with the disc-diffusion method were performed as recommended by Clinical and Laboratory Standards Institute (CLSI 2013) for the following antimicrobial agents: amikacin, cefepime, ceftazidime, ciprofloxacin, gentamicin, imipenem, meropenem, piperacillin/tazobactam and tobramycin. Colistin minimum inhibitory concentrations (MIC) were determined by broth microdilution (CLSI 2013). To calculate the resistance percentages, a single isolate was selected per patient. When several isolates were available from the same patient, we selected the isolate resistant to the greatest number of drugs. Resistance percentages were compared with those obtained in a previous study (Pellegrino et al. 2002).

*Carbapenemase production and carbapenemase-encoding genes* - All ceftazidime or imipenem resistant isolates were submitted to the MbL double-disc synergy test, as previously described (Picão et al. 2008). Isolates from infections were analysed by a modified CarbaNP test (Nordmann et al. 2012). In brief, cell lysis was performed by 30-min bath sonication instead of using commercial lysis buffer, and imipenem-cilastatin was used as substrate. Isolates exhibiting a positive MbL double-disc synergy test or modified CarbaNP test were screened for carbapenemase encoding genes (*bla*_IMP-type_, *bla*_VIM-type_, *bla*_SPM-1,_ *bla*_SIM-1,_ *bla*_GIM-1,_ *bla*_NDM-1_, *bla*_KPC_ and *bla*_GES-like_) by polymerase chain reaction (PCR) (Poirel et al. 2000, Hossain et al. 2004, Mendes et al. 2007). Distinction of the *bla*_GES_ type was achieved by gene sequencing.

*Detection of non-carbapenemase resistance mechanisms* - Mechanisms of carbapenem resistance other than carbapenemase production were investigated in carbapenem-resistant (n = 10) and carbapenem-susceptible (n = 25) isolates from infection episodes, including *oprD* gene mutations, overexpression of efflux systems and overproduction of AmpC-type β-lactamase.

The entire *oprD* gene (1412 bp) was sequenced as previously described (Rodríguez-Martinez et al. 2009b). OprD deduced amino acid sequences were compared with *P. aeruginosa* PAO1 (GenBank AAG04347.1) using Bioedit (v. 7.0.9.0) to investigate structural modifications (Hall 1999).

The MIC of imipenem and meropenem were determined by Etest (AB Biodisc, Solna, Sweden) and were compared with results obtained after incorporation of the efflux system inhibitor PAβN (50 μM) (Lomovskaya et al. 2001) and the AmpC inhibitor cloxacillin (250 mg/L) (Rodríguez-Martinez et al. 2009a) to the medium. In this comparison, at least two-fold dilution differences indicated efflux system overexpression or AmpC-type β-lactamase overproduction (Rodríguez-Martinez et al. 2009a).

*Detection of RmtD 16S rRNA methylase encoding gene* - Amplification of *rmtD* gene was performed as described (Corrêa et al. 2014) for the isolates from infection (n = 35) of the present study and the previously characterised aminoglycoside resistant isolates (n = 15) (Pellegrino et al. 2002).

*Strain typing* - Typing by randomly amplified polymorphic DNA PCR (RAPD-PCR) (Mahenthiralingam et al. 1996) was performed with the aim of screening for main clusters of types during the study. For the RAPD test, a subgroup of isolates from clinical infections and a sample from colonisations, including at least one isolate obtained during each week of admission per patient, were selected. When multiple isolates were available, we chose the one resistant to the higher number of antimicrobial agents. In addition, we performed RAPD typing for all 17 environmental isolates. DNA band patterns were analysed with Gel Compar software version 4.01 (Applied Maths, Kortrijk, Belgium), and isolates with 100% similarity were included in a single RAPD-type.

PFGE was performed for a smaller collection of isolates as a more robust and discriminatory method as pre-selection for multilocus sequence typing (MLST). The PFGE sub-group included isolates from infection and isolates from colonisation obtained immediately before the onset of infection. PFGE was performed as described (Pellegrino et al. 2002). Briefly, DNA plugs were digested with *SpeI* (10 U/isolate, Promega, Madison, US) at 37ºC; band patterns were analysed by Gel Compar and interpreted by visual inspection. Isolates with up to two band differences corresponding to 90% similarity were included in a single pulsotype.

For MLST, the selection included 11 clinical isolates from the predominant pulsotypes and 4 ICU environment isolates (one highly resistant and one susceptible isolate of a dominant RAPD-type, the single RAPD-type also found in a patient, and the *bla*_SPM-1_ carrying isolate). The MLST scheme available at University Warwick, UK, was performed with modifications (Curran et al. 2004). Adjustments were necessary due to several non-specific amplifications with primers proposed by these authors; thus, sequencing was not feasible. New primers were designed with primer-blast assistance (http://www.ncbi.nlm.nih.gov/tools/primer-blast) and used for both amplification and sequencing, with exception of *aroE* (Table I). A fresh bacterial suspension in water showing turbidity equivalent to MacFarland’s scale 0.5 was used as template DNA. PCR parameters were initial DNA denaturation at 96ºC for 10 min; 35 cycles of 96ºC for 1 min, the appropriate annealing temperature (Table I) for 30 s, and 72ºC for 1 min; and a final extension step at 72ºC for 7 min. PCR products were purified by ExoSAP-IT (Affymetrix, Inc., Santa Clara, CA) and submitted to direct sequencing at the DNA Sequencing Facility of Macrogen Korea (Geumcheoun-gu, Seoul, Korea). MLST clonal complexes (CC) were defined as ST with five or more identical alleles by eBURST analysis with PHYLOVIZ version 1.0 (Francisco et al. 2012). A previously characterised isolate was also typed: isolate PHU169 (*bla*_SPM-1_ positive and widespread among four hospitals in Rio de Janeiro, of RAPD-type “a” and PFGE-type “A”) (Pellegrino et al. 2002).

**TABLE I:** Oligonucleotides used for multilocus sequence typing

Gene target	Primer name	Sequence (5’ to 3’)	Amplicon size (bp)	Nt location	AT (ºC)	Reference
acsA	acsA-F	GCCACACCTACATCGTCTAT	520	935-1454bp	53	Curran et al. (2004)
	acsA-R	ACGAAGCGGTCATGGTC				Present work
*aroE*, amp	aroE-F	CACTCCGGCAAAGGAACGA	867	127-993bp	55	Present work
	aroE-R	CTCAAATGCCGCCTGACAAC				Present work
*aroE*, seq	aroE-F	TGATCCACCGCCTGTTCG	676	53-728bp	50	Present work
	aroE-R	ACCAGCATGCCCAGGC				Present work
guaA	guaA-F	CGACAAGGTCACCGAGATGC	652	432-1083bp	57	Present work
	guaA-R	GACGTTGTGGTGCGACTTGA				Present work
mutL	mutL-F	GCGACCTGTTCTTCAACAC	530	455-984bp	53	Present work
	mutL-R	GGTGCCATAGAGGAAGTCAT				Present work
nuoD	nuoD-F	TCGATCCCTACTCCCTGTCC	600	521-1120bp	53	Present work
	nuoD-R	CCAGCTTGTCCCAGCC				Present work
ppsA	ppsA-F	GGCCAAGCAGGCCAT	578	891-1468bp	50	Present work
	ppsA-R	GRTTGCCGACGTTCATCAT				Present work
trpE	trpE-F	ATCAAGGACTACATCCTGGC	727	697-1423bp	55	Present work
	trpE-R	TGATGGTTTCTTCCCACTCC				Present work

AT: annealing temperature; bp: base pairs; amp: amplification; seq: sequencing; Nt: nucleotide, referring to numbers of gene sequence of PAO1 (Genebank NC_002516.2).

*Statistical analysis* - Differences between the proportions were compared using Chi-square or Fisher’s exact tests. Differences were considered significant at p-values < 0.05.

## RESULTS

*Antimicrobial susceptibility, carbapenemase production and carbapenemase-encoding genes* - We tested all 465 isolates recovered for susceptibility to amikacin, cefepime, ceftazidime, ciprofloxacin, colistin, gentamicin, imipenem, meropenem, piperacillin-tazobactam and tobramycin. Table II presents the comparison of the non-susceptible isolates from the present study and those previously obtained (Pellegrino et al. 2002). The results for isolates of the present study were additionally interpreted using CLSI (NCCLS) 1999 breakpoints. Amikacin resistance was significantly reduced in the current study isolates (p = 0.01). ICU environment isolates included seven (41%) that were susceptible to all antimicrobials and a few resistant to other agents; the ciprofloxacin resistance rate (53%) was the highest. In total, 79 clinical and five environment isolates were resistant to ceftazidime or imipenem and were submitted to MβL production double-disc synergy test. A single environment isolate (CTI33) recovered from haemodialysis portable equipment had a positive MβL result.

**TABLE II:** Antimicrobial resistance rates among *Pseudomona aeruginosa* isolates of the present study and those previously described (Pellegrino et al. 2002)

Antimicrobial agent	Number and (%) of resistant isolates		

	Clinical isolates		Environmental isolates from present study (2007-2008) n = 17

	Present study (2007-2008) n = 88^a^	Pellegrino et al. (2002) (1999-2000) n = 115^b^	
Amikacin	16 (18.2)	41 (35.6)^*^	5 (29.4)
Cefepime	33 (37.5)	47 (41.0)	3 (17.6)
Ceftazidime	33 (37.5)	42 (36.5)	5 (29.4)
Ciprofloxacin	30 (34.1)	49 (43.0)	9 (52.9)
Gentamicin	30 (34.1)	55 (48.0)	7 (41.2)
Imipenem	31 (35.2) - 27 (30.7)^c^	44 (38.3)	4 (23.5)
Meropenem	24 (27.3) -20 (22.7)^c^	35 (30.4)	1 (5.9)
Piperacillin-tazobactam	32 (36.4) - 25 (28.4)^c^	42 (36.5)	0
Tobramycin	29 (33.0)	Not tested	8 (47.0)

One isolate with resistance to the greatest number of antimicrobial agents was selected per patient from a total of 225^a^ or 200^b^; all isolates were susceptible to colistin (minimum inhibitory concentration < 2 μg/mL). Data for interpretation with Clinical and Laboratory Standards Institute (NCCLS) 1999 are presented in ^c^; *: comparison between clinical isolates exhibited p = 0.01; other comparisons were p-values > 0.06.

We performed PCR for carbapenemase-encoding genes and the CarbaNP test in the sub-group of isolates including the 35 isolates from clinical infections (observed in 30 patients) and CTI33 (isolate from haemodialysis portable equipment). CTI33 was the single isolate carrying a carbapenemase-encoding gene, *bla*_SPM-1_, that exhibited a positive CarbaNP test. Four clinical isolates carried *bla*_GES-1_, a b-lactamase without carbapenemase activity.

*Detection of non-carbapenemase resistance mechanisms* - The 35 isolates from clinical infections, including 10 (29%) and 7 (20%) that exhibited imipenem and meropenem resistance, respectively, were selected for determination of carbapenem-resistance mechanisms.

Efflux overexpression, AmpC-type β-lactamase overproduction and *oprD* gene mutations were frequently observed, as described in Table III. Sequences of the *oprD* gene from imipenem-susceptible isolates were similar to that of the *P. aeruginosa* PAO1 strain. In addition, imipenem-resistant isolates had *oprD* gene inactivating mutations: a frameshift produced by deletions (n = 6; 60%) resulting in premature stop codons was the most frequent, followed by base pair substitutions (n = 3; 30%) and a 4-bp insertion (n = 1; 10%) in the coding sequence. Imipenem resistance was statistically associated with efflux overexpression and AmpC-type β-lactamase overproduction (p < 0.05) and completely matched *oprD* mutation. Meropenem resistance was statistically associated with efflux overexpression and *oprD* mutation (p < 0.05). AmpC-type β-lactamase overproduction was increased (57%) among resistant isolates compared with susceptible isolates (21%), but the difference was not significant (p = 0.1). The combinations of these carbapenem resistance mechanisms are presented in Table IV.

**TABLE III:** Resistance marker present in 35 *Pseudomonas aeruginosa* isolates according to carbapenem susceptibility

Resistance mechanism	Number and (%) isolates						

	Imipenem				Meropenem		
	
	R (10)	S (25)	p		R (7)	S (28)	p
Efflux system	6 (60)	1 (4)	0.001		4 (57)	3 (11)	0.03
AmpC	6 (60)	4 (16)	0.03		4 (57)	6 (21)	0.16
*oprD* mutation	10 (100)	0 (0)	< 0.001		7 (100)	3 (11)	< 0.001

R: resistant; S: susceptible.

**TABLE IV:** Mechanisms of carbapenem resistance and antimicrobial resistance among clinical *Pseudomonas aeruginosa* isolates from infection

Mechanisms of resistance: efflux system, AmpC, *oprD* mutation* (number of isolates)	Antimicrobial resistance profile** (number of isolates)	MIC*** (μg/mL)	

		IPM	MER
+,+,+ (3)	IPM MEM FEP CIP CAZ TZP TOB GEN (1)	32	32
	IPM MEM (1)	24	12
	IPM (1)	12	4
+,-,+ (3)	IPM MEM AMK FEP CIP CAZ TZP TOB GEN (1)	24	> 32
	IPM MEM FEP CIP CAZ TZP TOB GEN (1)	16	> 32
	IPM (1)	6	1.5
-,+,+ (3)	IPM MEM FEP CIP CAZ TZP TOB GEN (2)	24	> 32
		32	> 32
	IPM (1)	16	2
-,-,+ (1)	IPM MEM FEP CIP CAZ TZP TOB GEN (1)	4	6
+,+,-(1)	Susceptible (1)	1.5	0.38
-,+,- (3)	Susceptible (3)	1.5-0.75	0.19-0.38
-,-,- (21)	AMK FEP CIP CAZ TZP TOB GEN (2)	0.75-1.5	0.094-2
	AMK FEP CIP CAZ TOB GEN (2)	0.75	1
	AMK FEP CAZ PIP (1)	0.75	0.19
	TOB, GEN (1)	1	0.25
	Susceptible (15)	0.5-1	0.064-0.5

*: assessed by comparison of carbapenem minimum inhibitory concentration (MIC) and in combination with PAβN or cloxacillin and sequencing of *oprD*. Antimicrobial susceptibility was determined by disc diffusion (**) or E-test (***); + or - : presence or absence of mechanism of resistance, respectively; AMK: amikacin; CAZ: ceftazidime; CIP: ciprofloxacin; FEP: cefepime; GEN: gentamicin; IPM: imipenem; MEM: meropenem; TZP: piperacillin-tazobactam; TOB: tobramycin; IMP and MER MIC interpretation: ≤ 2, susceptible; 4, intermediate; ≥ 8, resistant.

*Detection of RmtD 16S rRNA methylase encoding gene* - The *rmtD* gene was not detected among isolates from infections, whereas seven (47%) of 15 clinical isolates of the previous study (Pellegrino et al. 2002) carried this gene. Five of these seven were *bla*_SPM-1_ positive, including isolate PHU169.

**Fig. 2:**
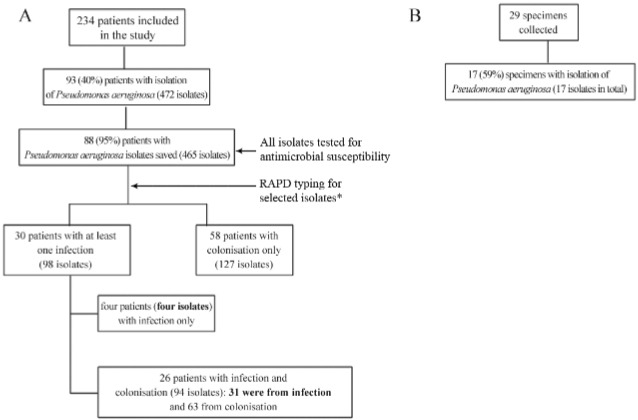
: temporal distribution of random amplified polymorphic DNA-types among *Pseudomonas aeruginosa* isolates from colonisation and infection.

*Strain typing* - A diverse clonal population was observed among the subgroup of 225 clinical isolates and was selected for typing (according to the criteria described in methods). A total of 106 (47%) isolates were included in 34 RAPD-types with clusters (each with 2-10 isolates), and 119 were unique genotypes. One clinical isolate was not typeable. Fig. 2 highlights the temporal distribution of RAPD types detected in at least four clinical isolates. The 17 ICU environment isolates were clustered in 10 RAPD types. One genotype recovered from a sink drain was indistinguishable from two clinical isolates; in addition, the RAPD-type of CTI33 was indistinguishable from that of the *bla*_SPM-1_ positive control isolate (PHU169).

A total of 33 isolates from infection, 22 isolates from colonisation and the ICU environment isolate CTI33 were typed using PFGE (two isolates from infection were PFGE-untypeable). Isolates were included in 37 PFGE-types: two with six isolates each, two with three isolates each, nine with two isolates each, and 24 were unique. Only the CTI33 had a band profile indistinguishable from the SPM-producing control isolate PHU169.

Isolate CTI33 and control PHU169 were the only ones included in ST277 (Table V). No clinical isolate belonged to this clone. Three new allele combinations were detected (ST1602, ST1603 and ST1844) for clinical and environmental isolates.

**TABLE V:** Characteristics of sequence-types (ST) detected among clinical and environmental study isolates

Isolate	Antimicrobial resistance Profile	ST	CC	Countries
Clinical Isolates				

PCI032	AMK, FEP, CIP, CAZ, TZP, TOB, GEN	1844	Singleton	Brazil
PCI042	MEM, FEP, CIP, IPM, CAZ, TZP, TOB, GEN	244	244	Australia, Central African Republic, China, France, Ivory Coast, Nigeria, Poland, Russia, Spain, UK
PCI045	Susceptible	235	235	Australia, Belarus, Brazil, Central African Republic, China, Croatia, France, Hungary, Ivory Coast, Kazakhstan, Nigeria, Norway, Poland, Republic of Belarus, Russia, Serbia, Singapore, Spain
PCI124	MEM, AMK, FEP, CIP, IPM, TZP, TOB, GEN			
PCI059	IPM	890	Singleton	Australia, Brazil
PCI073	Susceptible	1767	244	Brazil
PCI126	Susceptible	532	244	Australia, Brazil, France, Spain
PCI133	Susceptible	1027	SLV of 2223	Brazil
PCC457	MEM, FEP, CIP, IPM, CAZ, TZP, TOB, GEN	1768	244	Brazil
PCC524	FEP	1769	195	Brazil
PCC762	MEM, AMK, FEP, CIP, IPM, CAZ, TOB, GEN	1602	SLV of ST1937	Brazil

Environmental isolates				

CTI9.2B	AMK, CAZ, FEP, GEN, IPM, CIP, TOB	1602	SLV of ST1937	Brazil
CTI9.2A	AMK, CAZ, FEP, GEN, IPM, CIP, TOB	1603	244	Brazil
CTI19	Susceptible	446	244	Australia, Brazil, France, Spain
CTI33	CAZ, FEP, GEN, IPM, MEM, CIP, TOB	277	244	Australia, Austria, Brazil, China, Central African Republic, Spain

AMK: amikacin; CAZ: ceftazidime; CIP: ciprofloxacin; FEP: cefepime; GEN: gentamicin; IPM: imipenem; MEM: meropenem; TZP: piperacillin-tazobactam; TOB: tobramycin; SLV: single-locus variant.

## DISCUSSION

The present study aimed to investigate the fate of *P. aeruginosa* that is resistant to carbapenems due to SPM-1 production after its previously reported explosive emergence at a university hospital in Rio de Janeiro, Brazil (Pellegrino et al. 2002). A cohort of patients was thoroughly screened for *P. aeruginosa* infection and monitored for one year. No strain with an SPM-1 compatible phenotype was identified among 465 clinical isolates, as revealed by a negative MbL double-disc synergy test for all isolates exhibiting ceftazidime or imipenem resistance. It is still possible that the *bla*_SPM-1_ gene was present in a carbapenem- or ceftazidime-susceptible isolate; however, this resistance determinant has been rarely described in carbapenem-susceptible isolates (Pellegrino et al. 2008, Picão et al. 2012). We obtained a single *bla*_SPM-1_ carrying isolate from the environment. Apparently, SPM-1-producing isolates of the pandemic lineage ST277 were replaced by polyclonal *P. aeruginosa*. The temporal distribution of *P. aeruginosa* isolation and the predominant RAPD types revealed an endemic fluctuation of cases with no major clusters or types in the study. The subsequent scenario followed the globalisation trend and included the eventual circulation of international clones, as revealed by two and seven isolates of CC235 and CC244, respectively, previously detected in the five continents.

Antimicrobial resistance decreased in more recent isolates, with a statistically significant difference for amikacin. Data for isolates of the present study were additionally analysed according to the same breakpoints used for isolates of Pellegrino et al. (2002); the difference increased for carbapenems and piperacillin-tazobactam. These results are consistent with the decline of SPM-positive isolates and the absence of the *rmtD* gene in the more recent isolates.

The mechanism of carbapenem resistance was thoroughly characterised for a subset of isolates, including all obtained from infections. Carbapenemase production was absent, and resistant isolates displayed the classic carbapenem resistance determinants. Inactivating mutations in *oprD* gene described in previous studies (Xavier et al. 2010, Castanheira et al. 2014) were fully congruent with imipenem resistance and associated with higher MIC values when AmpC overproduction was present. Efflux pump overproduction was constantly detected in association with other carbapenem-resistance mechanisms. All three isolates exhibiting AmpC overproduction as the single resistance mechanism were b-lactam susceptible. In fact, AmpC overproduction is a gradual and complex phenomenon, and the occurrence of b-lactam resistance is unpredictable (Lister et al. 2009). This observation of the present study indicates the assay used had sufficient sensitivity, and the isolates did not have a wild-type AmpC-producing phenotype. The clinical implication of this result remains a challenge (Lister et al. 2009). The finding of carbapenem-susceptible isolates with efflux overexpression or AmpC overproduction reflects the notion that resistance in *P. aeruginosa* is mostly determined by multiple mechanisms, each with slightly decreasing susceptibility, depending on each strains’ genetic background (Castanheira et al. 2014).

We observed that the SPM-1 clone decreased in frequency when isolates obtained in two timeframes eight year apart were compared. This exchange could have been driven by the emergence of carbapenem-resistant *Acinetobacter baumannii* international clones in the study hospital, as previously reported (Martins et al. 2013), and of other resistant isolates, including *P. aeruginosa*. The study isolates were collected from 2007 to 2008, but we believe the SPM-1 clone was rare in the hospital until recently. A review of the surveillance data for multidrug-resistant pathogens from August 2013 to July 2014 revealed only two isolates with a colistin-only susceptible profile, suggestive of SPM-1 producing strains.

SPM-1 *P. aeruginosa* fading could also be explained by a decrease in bacterial fitness, as proposed by others to occur with highly antimicrobial resistant strains (Andersson & Hughes 2010). A better understanding of the mechanisms of emergency, spread and dissemination of pandemic clones is needed. As antimicrobial resistance becomes a global public health threat, this knowledge will be essential for the development of modern efficient strategies to overcome infections.
